# Navigating the current landscape of scientific publishing – the *Molecular Oncology* perspective

**DOI:** 10.1002/1878-0261.13271

**Published:** 2022-06-20

**Authors:** Kevin M. Ryan

**Affiliations:** ^1^ Molecular Oncology Editorial Office FEBS Press Cambridge UK; ^2^ Cancer Research UK Beatson Institute Glasgow UK; ^3^ Institute of Cancer Sciences University of Glasgow UK

With your experiments and analyses completed, thoughts turn to writing up your work and submitting for publication. This cycle has not changed for decades, but in recent years there has been an exponential increase in the number of journal options in any given field. So, how do you choose the best journal for you? Terms such as subscription journal, hybrid journal, open access journal and society journal are ones that we all hear, but what does it all mean? Within this short commentary, I highlight several aspects of publishing that are worth considering when choosing which journal to submit your work. The perspectives are admittedly written from the office of *Molecular Oncology*, but the comments also represent an objective view from an academic scientist, which could be applied to many areas of science.

## The historical perspective: Why do we publish?

In many ways, the reason why we publish our work is sometimes lost in the need to publish to graduate, the requirement to fulfil objectives for current grants and the necessity to have publications for future grants. It must be remembered, however, that the founding principle of scientific publication is to tell the community about your recent discovery! Within this, we desire that as many people as possible read our work, who will then hopefully recognise its merits and use the findings to build the next stage of scientific endeavour. It is on this basis that the most recognised journals in science have gained their esteem as, in a time before the Internet, they were the most accessible and read. By association, scientists wanted to publish their work in these journals to gain the most exposure, and this led to competition for space and the ability of journals to select what they perceived to be the most exciting discoveries of the time. During this time, journals were purchased either individually (*i.e*. by a scientist) or by an institution. This led to hierarchy in the accessibility of journals with some institutions being able to pay for the majority of available journals, whereas others were only able to acquire a few. This resulted in a situation where the dissemination of scientific discovery was not available to all and the assumption that important discoveries were only reported in the most widely purchased high‐profile journals. As we know, this is not true, with many discoveries that have led to Nobel prizes being published in journals that are not at the top of the ‘perceived’ pyramid of publishing. It was on this basis that the San Francisco Declaration of Research Assessment (DORA, https://sfdora.org) was established to recognise published science, not based on where it is published, but simply on its merits. Many universities, research institutes and publishers around the world have agreed with this principle, and are now signatories of DORA.

## A change in the landscape: Subscription, hybrid, open‐access and the society journal.

Building on the views of initiatives like DORA, many academics changed their view on how scientific literature should be accessed. There was a move from the traditional ‘pay to read’ to a new ‘pay to publish’ model. Under the ‘pay to publish’ model, the assumption is that published work would be accessible to all, particularly with the advent of the Internet. This ‘pay to publish’ model defined what is known as ‘open‐access publishing’. The onus in this model, however, is on the authors to pay larger fees than previously required, due to the lack of revenue from readers needing to purchase the journal. As a result, many traditional journals adopted a ‘hybrid’ model where some material is published open access for a higher fee (usually designated with the ‘open‐access fee’) and other material is published behind a ‘pay wall’ for a lower fee, in some cases with the article becoming freely available after a period of time.

Most scientists and funding bodies consider that the open‐access route is the best option for the global dissemination and recognition of their work. This is additionally supported by global and European initiatives like Plan S (https://www.coalition-s.org/) that aim at full and immediate open access of peer‐reviewed publications of all research funded by either public or private grants. However, as outlined above, open‐access publishing usually comes at an additional cost. As the funds of many laboratories and institutions are limited, avenues have been developed in many countries to deal with this additional financial toll. For example, in the case of the FEBS Press journal *Molecular Oncology* (https://febs.onlinelibrary.wiley.com/journal/18780261), the publisher Wiley have established transformational agreements with governing bodies in several countries https://www.wiley.com/network/archive/wiley-s-transformational-agreements-a-summary), so that open‐access fees are paid through the agreement rather than the scientific lab. This means that in some countries publishing ‘open access’ can be completely free of charge, at least from the lab's perspective. This does not, however, apply to all countries and so some journals, including all those published by FEBS Press (https://authorservices.wiley.com/open-research/open-access/for-authors/waivers-and-discounts.html), allow authors from a list of developing countries to apply for publication fee waivers.

## What do authors get for their publication fees – the society journal

It will not have escaped your attention that whether you choose to publish in a traditional subscription journal or in an open‐access journal, the fees for publishing are not trivial. As a result, it may have crossed your mind as to where all the money goes. In an era where we could simply post our work on a social media or pre‐print platform, it must be remembered that all journals offer varying levels of typesetting, copy editing and peer review, which all improve the final article but come at a cost to manage and implement. Peer review can often be seen as one of the most difficult parts of publishing. In my experience, the process of peer review should be perceived positively, akin to a service to improve the robustness and impact of published science, while equally returning work that is not acceptable for publication in its present form. While it is annoying when our papers are rejected, this has to be a clear facet of reputable journals that support science by recognising progress and supporting credibility. The key factor in peer review, however, is being asked to address what is necessary to convey the message of the discovery rather than what is beyond the scope of the study. This balance is no better achieved by journals that are run by active research scientists, who at first hand understand that the study should be solid and reproducible, but this does not necessarily require the generation of several new transgenic models!

Having established that certain aspects of publishing come at a cost, but are worthy, is this the whole story for those publication fees? Well, of course not! Publishing is a business and as with all businesses, there needs to be profit. So, the question then is, where does this profit go? With many journals, this goes to the investors and/or shareholders of the journal or the journal’s parental publishing company. As a pushback to this situation, many scientific organisations or foundations decided to establish their own journals, termed ‘Society Journals’, where the profits from publishing go back into the associated organisation or community that they serve. For example, in the case of *Molecular Oncology* and all other FEBS Press journals, all profits go to the Federation of European Biochemical Societies (FEBS), which provides funds for meetings, lab exchanges, fellowships and other initiatives that promote the careers of young biomedical scientists. So, the question when choosing a journal to submit your paper to is often first based on the reputation and standing of the journal, but it should then also come down to whether you feel you really need to send your work to a non‐society journal or whether a society journal may be an equal, or even a better option. Even when removing my admitted bias as Editor‐in‐Chief of *Molecular Oncology*, it would seem that publishing in a society journal should always be a consideration.

## Other considerations: What else does the journal do for you and your field?

**Fig. 1:**
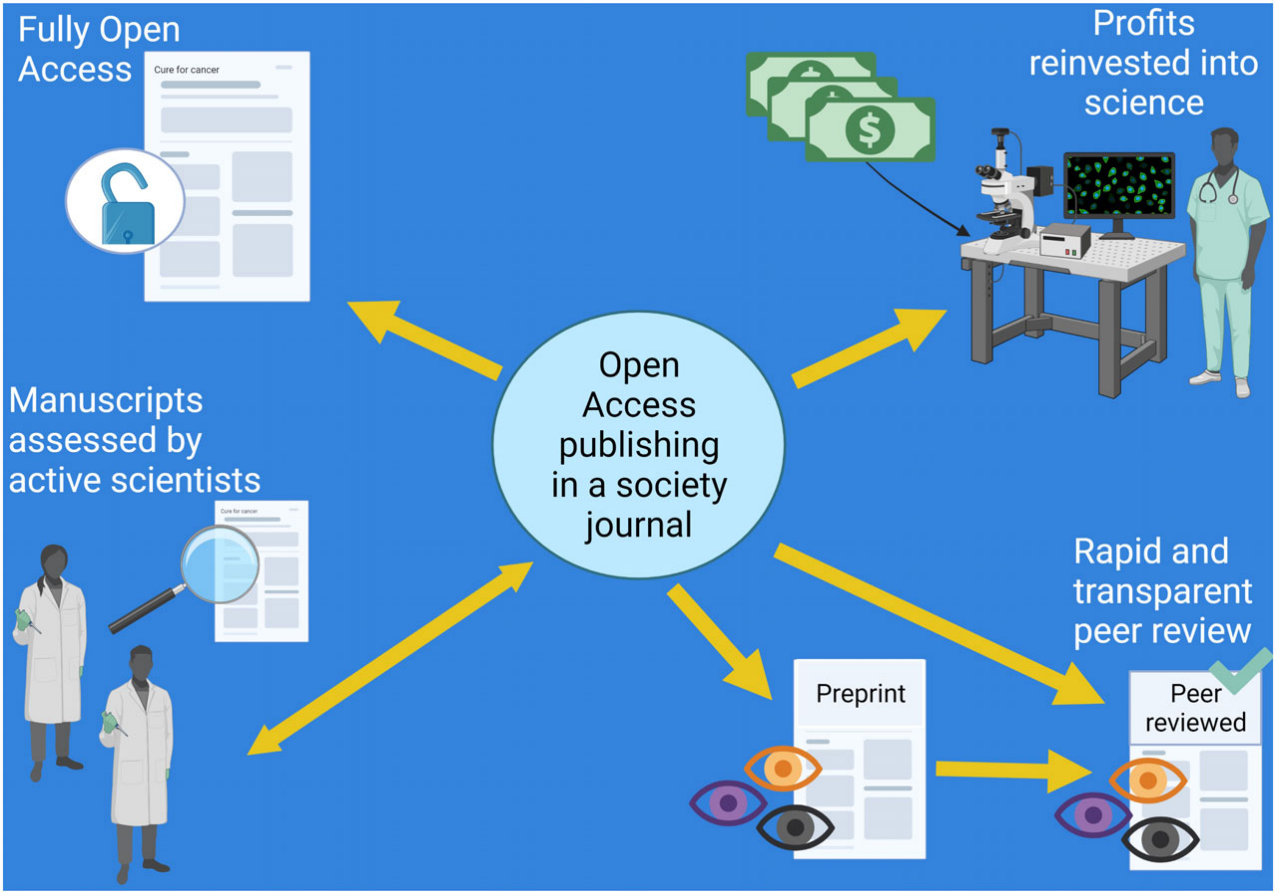
*Molecular Oncology* as a model of an open‐access society journal. Articles submitted to *Molecular Oncology* are initially assessed and further handled by scientists actively working within the scope of the journal. Direct submissions or manuscripts pre‐existing as preprints are streamlined through a rapid and transparent review process by experts in the field. Once an article has been accepted in a society journal such as *Molecular Oncology* and publication fees have been adjusted according to existing transformational agreements, it is published as fully open access. The related publication fees are transferred to the underlying society (*e.g*. FEBS in the case of *Molecular Oncology*), and reinvested to contribute to and promote the advancement of research and education for the public benefit in the sciences. [Colour figure can be viewed at wileyonlinelibrary.com]

It might be assumed that all we need to consider when choosing a journal in which to publish is the exposure gained, the fee, the choice of open access and whether it is a society journal (Fig. 1). However, many journals, especially the society journals, are working in other ways to improve and be of benefit to the scientific community. Many journals will issue editorials and commentaries relating to recent developments published in the field. Some journals, and again using *Molecular Oncology* as an example, also publish articles relating to science policy. To do this, we have a long‐standing connection with the European Academy of Cancer Sciences (EACS) and have published several articles reporting the latest developments at EACS and how these affect the cancer community in Europe and beyond. More recently, we have also partnered with the European Association for Cancer Research (EACR) to reciprocally share the dissemination of our initiatives and events. As part of this, if you are member of EACR (www.eacr.org), *Molecular Oncology* are also offering a 10% discount in publication fees! Moreover, this saving in publication fees is greater than the cost of joining EACR for those who are not already members!

In summary, I hope that this Editorial helps to navigate the current, often bewildering, choices in science publishing and will guide authors towards the conscious decision on where to submit their manuscripts. Lastly, the team at *Molecular Oncology* and I hope that if you work in any aspect of cancer research from lab to clinic, you will consider submitting your next manuscript to *Molecular Oncology*!

